# Association of Sleep Duration and Physical Activity with Healthy Eating Habits Among Peruvian Secondary School Students

**DOI:** 10.3390/nu18172775

**Published:** 2026-08-25

**Authors:** Jennyfer A. Remón-López, Raquel Chilón-Llico, Gutember Peralta-Eugenio, Wilter C. Morales-García, Liset Z. Sairitupa-Sanchez, Sandra B. Morales-García, Oriana Rivera-Lozada, Mardel Morales-García

**Affiliations:** 1 Escuela de Nutrición Humana, Facultad de Ciencias de la Salud, Universidad Peruana Unión, Lima 15102, Peru; 2 Facultad de Ciencias de la Salud, Escuela de Psicología, Universidad Cesar Vallejo, Chimbote 02801, Peru; gperaltae@ucvvirtual.edu.pe; 3 Facultad de Teología, Universidad Peruana Unión, Lima 15102, Peru; wiltermorales@upeu.edu.pe; 4 Escuela de Psicología, Universidad Señor de Sipán, Chiclayo 14001, Peru; 5 Escuela de Medicina Humana, Universidad Señor de Sipán, Chiclayo 14001, Peruriveraoriana@uss.edu.pe (O.R.-L.); 6 Dirección de Investigación, Escuela de Posgrado, Universidad Peruana Unión, Lima 15102, Peru; mardelmorales@upeu.edu.pe

**Keywords:** sleep duration, physical activity, healthy eating habits, adolescents, Peru

## Abstract

**Background/Objectives:** Sleep duration and physical activity are key behavioral components in the development of healthy lifestyles during adolescence. In Peru, a considerable proportion of school students do not meet recommended levels of physical activity and may also experience insufficient sleep, which could negatively affect their eating habits. This study aimed to analyze the association between sleep duration, physical activity level, and healthy eating habits among Peruvian secondary school students. **Methods:** An observational, analytical, cross-sectional study was conducted among 275 secondary school students aged 12 to 17 years. Sleep duration, physical activity, and eating habits were assessed using validated questionnaires. Associations were analyzed using Pearson’s chi-square test and multivariable binary logistic regression. **Results:** Students who slept 7 h had higher odds of having healthy eating habits compared with those who slept 6 h or less (aOR = 2.525; 95% CI: 1.181–5.536; *p* = 0.018). In addition, students with low levels of physical activity showed lower odds of having healthy eating habits compared with those with high levels of physical activity (aOR = 0.097; 95% CI: 0.005–0.564; *p* = 0.032). **Conclusions:** Sleep duration and physical activity were associated with healthy eating habits among Peruvian secondary school students. These findings support the importance of promoting integrated school-based strategies that jointly address sleep, physical activity, and nutrition during adolescence.

## 1. Introduction

In recent years, the overall health of children and adolescents has gained increasing relevance in public health research due to the role that lifestyles play in physical, cognitive, emotional, and social development. Adolescence is a key stage for establishing health patterns that may persist into adulthood, since routines related to rest, diet, movement, and the use of digital technologies are consolidated during this period [1,2]. In this context, sleep duration, physical activity, and eating habits are interrelated behaviors that decisively influence the present and future well-being of school students [3,4,5,6]. Available evidence indicates that inadequate sleep patterns in school-aged populations are associated with greater adiposity, poorer academic performance, behavioral problems, and impaired daytime functioning [7,8,9]. Likewise, regular physical activity plays a protective role against multiple adverse health outcomes and contributes to emotional and metabolic balance during adolescence [10,11]. More recently, the literature has reinforced the need to understand these behaviors as part of an integrated system of health-related behaviors rather than as isolated factors [1,4,12].

The relationship between sleep and physical activity appears to be bidirectional. On the one hand, greater participation in moderate or vigorous physical activity may promote better sleep quality and duration; on the other hand, better sleep may increase the physiological and behavioral disposition to remain physically active [13,14,15]. Along these lines, recent evidence has shown that longer sleep duration is associated with a higher likelihood of engaging more frequently in physical activity among adolescents, suggesting that nighttime rest may act as a behavioral facilitator of more active lifestyles [16]. Complementarily, international recommendations continue to emphasize that a substantial proportion of adolescents do not meet the minimum levels of physical activity required to obtain substantial health benefits, a situation that reinforces the urgency of studying these behaviors jointly [1].

Diet is another central component of a healthy lifestyle that adds to this interaction. Insufficient sleep has been documented to be commonly associated with less healthy eating patterns, including higher consumption of ultra-processed products, sugar-sweetened beverages, and energy-dense foods, which increase the risk of overweight and obesity [17,18,19,20]. In turn, physical activity may contribute to the regulation of physiological mechanisms related to appetite and energy metabolism, and it may also be associated with more favorable food choices, such as greater consumption of fruits and vegetables and a lower preference for foods with low nutritional value [21,22,23]. Recent studies have reinforced this interdependence by showing that healthier dietary patterns and higher levels of physical activity are linked to better sleep quality and duration in adolescents [4,24]. Convergently, healthier eating habits, such as frequent consumption of breakfast, fruits, and vegetables, as well as lower intake of sweets and soft drinks, have also been associated with fewer sleep difficulties in children and adolescents [6].

Consequently, sleep, physical activity, and diet may form either a virtuous cycle or, conversely, a pattern of cumulative risk. When these behaviors are unfavorable, their effects tend to reinforce one another: insufficient sleep may increase daytime fatigue, reduce participation in physical activity, and promote less healthy food choices; in turn, sedentary behavior and an inadequate diet may further impair sleep quality [12,25,26]. This pattern may be worsened by contextual factors typical of contemporary school life, such as academic pressure, excessive homework, prolonged screen time, and exposure to electronic devices before bedtime, all of which have been associated with alterations in nighttime rest, lower levels of physical activity, and other unfavorable health indicators [2,27,28]. This point is especially relevant in the current context of digitalization, in which screen time, social media use, and sedentary habits may simultaneously modify adolescents’ sleep, movement, and eating routines [2,12,28].

In the Peruvian context, this issue takes on particular relevance. Several studies have shown that a significant proportion of school students and adolescents do not reach the recommended levels of physical activity, while sedentary behavior and prolonged screen time continue to increase [29,30,31]. This situation compromises not only cardiometabolic health but also rest, physical performance, and overall well-being [30,32,33,34]. In Peruvian school students, a high frequency of sleep disturbances has been reported, along with an association between physical activity and sleep problems, reinforcing the need to examine these behaviors in adolescent populations [4]. In addition, reduced sleep may decrease the energy available for engaging in physical activity, thereby reinforcing sedentary patterns and increasing vulnerability to unhealthy eating behaviors [4,29].

From a public health perspective, understanding this behavioral triad is essential, since the coexistence of insufficient sleep, physical inactivity, and inadequate eating habits during adolescence may promote risk trajectories that extend into adulthood, increasing the likelihood of noncommunicable chronic diseases [12,35]. Therefore, health promotion strategies in school settings should adopt an integrated approach aimed not only at encouraging exercise and healthy eating but also at improving sleep routines and reducing factors that interfere with rest. Indeed, recent international frameworks emphasize the need to implement school- and community-based interventions that jointly address movement behaviors, the food environment, and other determinants of adolescent well-being [1]. In this regard, examining how sleep duration and physical activity level are associated with healthy eating habits among Peruvian school students may provide useful evidence for designing more precise and context-sensitive interventions. Therefore, the objective of the present study was to analyze the association between sleep duration, physical activity level, and healthy eating habits among Peruvian secondary school students, considering relevant sociodemographic and anthropometric variables.

## 2. Materials and Methods

### 2.1. Study Design

An observational, analytical, cross-sectional study was conducted to evaluate the association between sleep duration, physical activity level, and eating habits among Peruvian secondary school students.

### 2.2. Participants and Sample Size

The study population consisted of secondary school students from three public educational institutions located in South Lima, Peru. Preliminary administrative coordination with the participating educational institutions began in November 2023; however, no student data were collected before ethics approval was granted. The actual data collection with participants was conducted between December 2023 and January 2024, after approval by the Ethics Committee. Students enrolled from the first to the fifth year of secondary school, of Peruvian or foreign nationality, who regularly attended classes and whose parents or legal guardians provided informed consent, as well as students who provided informed assent, were included. Incomplete questionnaires or those with inconsistent response patterns that prevented the valid classification of the main study variables were excluded. For operational purposes, an incomplete questionnaire was defined as any record lacking information on one or more variables required to classify sleep duration, physical activity level, or eating habits. Likewise, inconsistent response patterns were defined as records with contradictory answers within the same section of the questionnaire, uniform responses without variability across all items, or implausible values in anthropometric or behavioral variables that prevented the classification of the main variables.

The sample size was estimated using G*Power version 3.1.9.7, considering a binary logistic regression model with multiple explanatory variables, an expected odds ratio of 1.5, an event probability under the null hypothesis of 0.30, a significance level of 0.05, and a statistical power of 80% [36]. Under these assumptions, a minimum required sample size of 242 participants was obtained. Finally, 275 school students were included, exceeding the calculated minimum sample size and helping compensate for potential losses due to nonresponse or invalid records.

### 2.3. Procedure

The study was approved by the Ethics Committee of Universidad Peruana Unión (code: 2023-CE-FCS-UPeU-N°141). Subsequently, formal authorization was obtained from the authorities of the participating educational institutions. Before data collection, informed consent was requested from parents or legal guardians through a virtual form, and this information was also communicated through the students’ school diaries. Data were collected using a self-administered questionnaire completed by students during school hours under the supervision of the research team and the authorities of the educational institutions. Before completing the questionnaire, students received the informed assent form in the first section of the instrument, which explained the voluntary nature of their participation, the confidentiality of the information, and their right to withdraw from the study at any time without any consequences. Data collection was conducted anonymously, without recording contact information or any data that could identify the participants. The entire process was carried out in accordance with the ethical principles of the Declaration of Helsinki.

### 2.4. Instruments

*Sociodemographic questionnaire:* A questionnaire developed for this study was used to collect information on sex, age, school grade, body mass index-for-age, height-for-age, socioeconomic level, and hours of sleep. Sex was recorded as female or male, while age was reported in completed years. School grade was categorized as first, second, third, fourth, and fifth year of secondary school. Socioeconomic level was grouped into categories B, C, D, and E. Finally, hours of sleep were self-reported by the students and categorized into three groups: 6 h or less, 7 h, and 8 h or more. Body weight was measured using a Personal Scale digital scale, and height was measured using a mobile stadiometer endorsed by the National Institute of Health. Based on these measurements, body mass index was calculated using the formula weight/height^2^ and expressed in kg/m^2^. Since the participants were adolescents aged 12 to 17 years, nutritional status was classified using body mass index-for-age according to the cutoff points recommended by the Technical Guide for the Anthropometric Nutritional Assessment of Adolescents issued by the Ministry of Health of Peru and the National Institute of Health [37]. According to these criteria, thinness was classified as BMI-for-age <−2 SD, normal weight as ≥−2 SD and ≤+1 SD, overweight as >+1 SD and ≤+2 SD, and obesity as >+2 SD. For the analysis, the overweight and obesity categories were grouped as excess weight. Height-for-age was classified as short stature when it was <−2 SD and normal when it was ≥−2 SD [37].

*Eating habits:* Eating habits were assessed using the questionnaire employed by Sayán [38] in Peruvian school students. The instrument provides a global index of eating habits based on 26 items organized into two components: eating behavior and food consumption. The first component evaluates general practices related to daily meals, breakfast, snacks, and routines during eating; the second evaluates the frequency of consumption of various food groups, including healthy foods and energy-dense products. In the original study, the instrument showed acceptable internal consistency, with a Cronbach’s alpha of 0.747. The total score ranges from 26 to 130 points and classifies eating habits as healthy (97–130), somewhat unhealthy (62–96), and unhealthy (26–61). For the inferential analysis, the index was dichotomized into the presence of healthy eating habits versus absence of healthy eating habits.

*Physical activity:* Physical activity level was assessed using the Physical Activity Level Questionnaire validated by Gómez Campos et al. [39] in Peruvian adolescent school students. The instrument consists of 11 items that assess habitual physical activity performed over one week, considering activities carried out at school, during leisure time, while commuting, and in sports, recreational, or household activities. The questionnaire evaluates four main indicators: type of physical activity, frequency, duration, and intensity. In the original study, the instrument showed adequate reliability and reproducibility indicators, with Cronbach’s alpha values ranging from 0.97 to 0.98. For the present study, the total score was classified into three levels of physical activity: low, moderate, and high.

### 2.5. Statistical Analysis

Initially, a descriptive analysis of the sociodemographic, anthropometric, and behavioral variables of the sample was performed. Categorical variables were summarized using absolute and relative frequencies. Subsequently, a bivariate analysis was conducted to explore the association between healthy eating habits and the independent variables of the study. Pearson’s chi-square test was used for this purpose.

Because the dependent variable was dichotomous, binary logistic regression was applied to estimate crude odds ratios and 95% confidence intervals for the factors associated with the presence of healthy eating habits. Variables with a *p*-value < 0.25 in the bivariate analysis were considered candidates for the multivariable model in order to avoid the premature exclusion of potentially relevant variables. Subsequently, a multivariable binary logistic regression model was fitted to estimate adjusted odds ratios and 95% confidence intervals.

The final model included sex and school grade as covariates, in addition to the main exposure variables: sleep duration and physical activity level. Thus, the independent association of sleep duration and physical activity with healthy eating habits was assessed while controlling for relevant sociodemographic characteristics.

Multicollinearity among the independent variables included in the multivariable model was assessed using the adjusted variance inflation factor, with values below 3 considered indicative of the absence of relevant collinearity. In addition, model goodness-of-fit was evaluated using the Hosmer–Lemeshow test, and discriminative ability was assessed using the area under the ROC curve.

A two-tailed significance level of *p* < 0.05 was adopted. All analyses were performed using R software, version 4.1.1.

## 3. Results

### 3.1. Sample Characteristics and Bivariate Analysis

The sample consisted of 275 secondary school students aged 12 to 17 years. No missing values were identified in the main study variables; therefore, no participants were excluded due to incomplete questionnaires or missing data in the analyzed variables. Likewise, no records with inconsistent response patterns that prevented the classification of the main study variables were identified. Of the total participants, 48 students (17.5%) were classified as having healthy eating habits, whereas 227 (82.5%) showed an absence of healthy eating habits. In the total sample, females predominated (55.6%), as did students in the third year of secondary school (33.8%), those with normal BMI (53.8%), normal height-for-age (93.8%), and socioeconomic level C (41.5%). Regarding behavioral variables, 48.0% of the students reported sleeping 6 h or less, 31.6% slept 7 h, and 20.4% slept 8 h or more. In terms of physical activity, 63.3% had a moderate level, 19.6% had a high level, and 17.1% had a low level. In the bivariate analysis, statistically significant differences were observed according to sex, hours of sleep, and physical activity level. The proportion of healthy eating habits was higher among males than among females (60.4% vs. 39.6%; *p* = 0.021). Likewise, students who slept 7 h showed a higher proportion of healthy eating habits compared with those who slept 6 h or less (47.9% vs. 31.3%; *p* = 0.016). Similarly, students with a low level of physical activity had a lower proportion of healthy eating habits compared with those with high or moderate physical activity levels (2.1% vs. 29.2% and 68.8%, respectively; *p* = 0.005). No statistically significant differences were found according to school grade, age, BMI, height-for-age, or socioeconomic level (Table 1).

### 3.2. Unadjusted Logistic Regression Analysis

In the unadjusted logistic regression analysis, male sex showed higher odds of having healthy eating habits compared with female sex; however, this association did not reach statistical significance (OR = 1.817; 95% CI: 0.895–3.762; *p* = 0.101). Regarding school grade, students in the fifth year of secondary school had lower odds of having healthy eating habits compared with those in the first year of secondary school (OR = 0.066; 95% CI: 0.004–0.862; *p* = 0.044). However, the remaining school-grade categories did not show statistically significant associations. Neither BMI, height-for-age, nor socioeconomic level was significantly associated with healthy eating habits. In contrast, sleeping 7 h was associated with higher odds of having healthy eating habits compared with sleeping 6 h or less (OR = 2.652; 95% CI: 1.196–6.033; *p* = 0.018). Likewise, a low level of physical activity was associated with lower odds of having healthy eating habits compared with a high level of physical activity (OR = 0.103; 95% CI: 0.005–0.630; *p* = 0.040) (Table 2).

### 3.3. Multivariable Logistic Regression Model

In the multivariable binary logistic regression model, sleeping 7 h was significantly associated with a higher likelihood of having healthy eating habits compared with sleeping 6 h or less (aOR = 2.525; 95% CI: 1.181–5.536; *p* = 0.018). Likewise, having a low level of physical activity was associated with a lower likelihood of having healthy eating habits compared with having a high level of physical activity (aOR = 0.097; 95% CI: 0.005–0.564; *p* = 0.032). In contrast, male sex, sleeping 8 h or more, having a moderate level of physical activity, and being in higher school grades did not show statistically significant associations with the outcome. The adjusted VIF values ranged from 1.015 to 1.050, indicating the absence of relevant multicollinearity among the variables included in the model. In addition, the Hosmer–Lemeshow test showed adequate model fit (χ^2^ = 4.951; *df* = 8; *p* = 0.763). The final model showed a significantly better fit than the null model (χ^2^ = 25.205; *p* = 0.003) and acceptable discriminative ability, with an area under the ROC curve of 0.714 (Table 3).

## 4. Discussion

The present study analyzed the association between sleep duration, physical activity level, and healthy eating habits among Peruvian secondary school students. The main findings showed that sleeping 7 h, compared with sleeping 6 h or less, was associated with a higher likelihood of having healthy eating habits. Likewise, a low level of physical activity was related to a lower likelihood of having such habits. In contrast, sex and school grade did not remain statistically significant in the multivariable logistic regression model. Taken together, these results support the idea that behaviors related to sleep, movement, and diet do not occur in isolation but rather form part of an interdependent behavioral pattern during adolescence [2,3,12,31,32,40].

Although differences according to sex were observed in the bivariate analysis, this association was not maintained in the multivariable logistic regression model. Therefore, the results suggest that sex, by itself, does not constitute a sufficiently robust independent factor to explain the presence of healthy eating habits in this sample. This finding is relevant because it avoids overemphasizing demographic differences that may be mediated or confounded by other factors related to the school, family, or behavioral environment. Rather than interpreting sex as a direct determinant, the data suggest that variability in eating habits may depend more on the interaction between daily routines, food availability, physical activity level, rest patterns, and exposure to unhealthy digital or food environments. This interpretation is consistent with recent studies indicating that health behaviors in adolescents are influenced by family, school, social, and environmental factors beyond strictly biological differences between males and females [23,41,42,43].

Regarding school grade, a tendency toward lower odds of healthy eating habits was observed among students in higher grades; however, this association did not reach statistical significance in the multivariable logistic regression model. Although this result should be interpreted with caution, it is plausible that progression through school is accompanied by changes in personal autonomy, academic demands, screen use, exposure to social media, and peer influence, all of which may modify food choices during adolescence [23,44,45]. Likewise, increased academic stress may promote less healthy eating patterns, particularly in contexts where energy-dense, low nutritional value foods predominate as coping strategies [46,47,48]. Similarly, peer-group influence may consolidate unhealthy eating practices during secondary school, especially when these practices become part of shared routines both within and outside the school environment [49]. Nevertheless, since this effect did not remain significant after adjustment, this finding should be considered exploratory.

One of the central findings of the study was the association between sleeping 7 h and a higher likelihood of having healthy eating habits compared with sleeping 6 h or less. This result is consistent with the literature documenting a relationship between shorter sleep duration and less favorable eating patterns in children and adolescents [4,6,7]. One possible explanation is that insufficient sleep alters neuroendocrine processes involved in appetite regulation and food reward. In particular, sleep restriction has been associated with alterations in appetite-related hormones, greater feelings of hunger, and a stronger preference for highly palatable and energy-dense foods [48,50,51]. Although some of this mechanistic evidence comes from classic studies, it remains relevant for explaining the physiological pathways linking insufficient sleep, appetite, and food choice.

In addition, insufficient sleep may lead to daytime fatigue, lower self-regulation, and greater impulsivity, conditions that may facilitate less healthy food choices. In the school context, these alterations may translate into greater consumption of snacks, sugar-sweetened beverages, or ultra-processed foods, especially when there are time constraints, accumulated fatigue, academic stress, or high exposure to unhealthy food environments [20,52,53]. This link may also be reinforced by prolonged screen use, since device screen time is often associated with less rest, greater sedentary behavior, and exposure to advertising or food-related stimuli of low nutritional value [2,12,28].

Other mechanisms may also be involved in the relationship between insufficient sleep and unhealthy eating. Shorter sleep duration may increase emotional eating, especially when adolescents use food as a strategy to cope with stress, fatigue, or emotional distress. Likewise, sleep restriction may intensify the hedonic response to highly palatable foods, strengthening the preference for sweet, salty, and energy-dense products. In addition, social factors such as peer influence, family routines, food availability in the school environment, and exposure to unhealthy food settings may shape food choices during adolescence [48,49]. Therefore, the relationship between sleep and eating habits should be interpreted not only through neuroendocrine mechanisms but also through emotional, behavioral, and social pathways.

Conversely, better nighttime rest promotes states of alertness and behavioral regulation that may facilitate healthier food decisions [4,6,54]. However, this finding requires careful interpretation. The fact that, in this sample, sleeping 7 h was associated with better eating habits than sleeping 6 h or less should not automatically be interpreted as a universal optimal sleep threshold for adolescents. Rather, the results indicate that, within the categories analyzed, sleeping 7 h was related to a more favorable eating profile than that of students who reported a shorter sleep duration. Therefore, the main value of this finding lies in showing the possible adverse effect of insufficient sleep, rather than establishing a definitive normative threshold. This clarification is important to avoid overinterpretation and to maintain coherence with the observational and cross-sectional nature of the study.

The second relevant finding was the association between low physical activity level and a lower likelihood of having healthy eating habits. This result is consistent with previous evidence suggesting that physical inactivity often coexists with other unhealthy behaviors during adolescence [12,55,56]. From a physiological perspective, regular physical activity contributes to the regulation of appetite, energy metabolism, and insulin sensitivity, processes that may promote a more adaptive relationship with food [57,58]. From a behavioral perspective, physically active adolescents may develop greater awareness of their health and, consequently, adopt other beneficial behaviors more frequently, such as choosing foods of better nutritional quality. This approach is consistent with self-efficacy theory, according to which perceived control over one’s own behaviors promotes the adoption and maintenance of healthy behaviors [59], as well as with the theory of planned behavior, which highlights the role of perceived behavioral control in making healthy choices [60]. Likewise, the school environment may amplify this association, since participation in sports and group activities may reinforce health-promoting norms and practices [2,55,61].

In broader terms, the study findings support the notion of clustering of healthy and unhealthy behaviors during adolescence. In other words, short sleep duration, low physical activity, increased screen time, and inadequate diet may form part of the same risk profile, reinforced by individual, family, school, and environmental factors [2,3,12,31]. This perspective is especially relevant in urban Latin American contexts, where inequalities in infrastructure, access to recreational spaces, availability of healthy foods, academic pressure, and screen exposure may simultaneously influence several dimensions of lifestyle. Consequently, addressing a single behavior in isolation may be insufficient if the interactions among sleep, physical activity, diet, and digital sedentary behavior are not considered.

### 4.1. Implications

The findings of this study have relevant implications for professional practice, public health, and research on lifestyles among Peruvian school students. At the applied level, the results suggest that the promotion of healthy eating habits in adolescents should not be addressed in isolation but rather in coordination with other daily behaviors, especially sleep duration and physical activity level. In this regard, school-based health promotion programs could incorporate a comprehensive approach that combines nutrition education, promotion of movement, and basic sleep hygiene strategies. Likewise, health and education professionals could play a key role in the early identification of risk patterns, as well as in the design of preventive actions aimed at strengthening healthier routines from early stages of development.

From a public policy perspective, these results support the need to strengthen school environments that simultaneously promote healthy eating, greater opportunities for physical activity, and better rest habits. This could translate into more integrated school-based interventions, including adequate spaces for physical activity, educational actions aimed at students and families, and strategies designed to reduce sedentary behaviors and routines that interfere with sleep. In resource-limited contexts, these actions could help reduce the early accumulation of risk factors linked to noncommunicable chronic diseases.

At the theoretical level, this study provides empirical evidence for understanding health behaviors as interrelated phenomena during adolescence. Rather than considering sleep, physical activity, and diet as independent domains, the results support an integrative perspective in which these behaviors tend to cluster and influence one another. This approach may be useful for future research aimed at exploring broader explanatory mechanisms, including psychological, family, school, and contextual factors that contribute to the consolidation of healthy lifestyles in adolescent populations.

### 4.2. Limitations

This study has several limitations that should be considered when interpreting the findings. First, due to its cross-sectional design, it is not possible to establish causal relationships among sleep duration, physical activity, and eating habits. Therefore, the results should be understood in terms of association rather than causality. Second, the information was collected using self-report instruments, which may introduce recall bias, underreporting, or social desirability bias. Future research should incorporate more objective measures, such as accelerometry for physical activity or sleep-monitoring devices.

Third, the sample consisted only of students from three public educational institutions in South Lima, which limits the generalizability of the results to other regions of the country, as well as to private or rural contexts. In addition, although the study included relevant sociodemographic and anthropometric variables, it did not consider other factors that could influence eating habits, such as overall diet quality, screen time, mental health, family environment, or academic stress. Consequently, future studies should adopt longitudinal designs, include more diverse samples, and consider a broader set of variables to more fully understand the interaction among these health behaviors.

## 5. Conclusions

This study provides evidence on the association between sleep duration, level of physical activity, and healthy eating habits among Peruvian secondary school students. The findings show that sleeping 7 h, compared with sleeping 6 h or less, and having a low level of physical activity were associated, respectively, with a higher and a lower likelihood of reporting healthy eating habits. Taken together, these results reinforce the importance of addressing adolescent nutrition within a broader framework of interrelated health behaviors. In this sense, health promotion strategies aimed at school students may benefit from an integrated approach that simultaneously considers sleep, physical activity, and diet as complementary components of well-being.

## Figures and Tables

**Table 1:** Sample Characteristics According to Healthy Eating Habits in Secondary School Students.

Characteristic	Total *n* (%)	Absence of Healthy Eating Habits *n* (%)	Healthy Eating Habits *n* (%)	*p*
Sex				0.021 *
Female	153 (55.6)	134 (59.0)	19 (39.6)	
Male	122 (44.4)	93 (41.0)	29 (60.4)	
School grade				0.5
1st year of secondary school	49 (17.8)	37 (16.3)	12 (25.0)	
2nd year of secondary school	47 (17.1)	40 (17.6)	7 (14.6)	
3rd year of secondary school	93 (33.8)	75 (33.0)	18 (37.5)	
4th year of secondary school	51 (18.5)	44 (19.4)	7 (14.6)	
5th year of secondary school	35 (12.7)	31 (13.7)	4 (8.3)	
Age (years)				>0.900
12	16 (5.8)	12 (5.3)	4 (8.3)	
13	42 (15.3)	33 (14.5)	9 (18.8)	
14	58 (21.1)	49 (21.6)	9 (18.8)	
15	87 (31.6)	72 (31.7)	15 (31.3)	
16	49 (17.8)	41 (18.1)	8 (16.7)	
17	23 (8.4)	20 (8.8)	3 (6.3)	
BMI				0.3
Underweight	2 (0.7)	1 (0.4)	1 (2.1)	
Excess weight	125 (45.5)	106 (46.7)	19 (39.6)	
Normal	148 (53.8)	120 (52.9)	28 (58.3)	
Height-for-age				0.3
Normal	258 (93.8)	211 (93.0)	47 (97.9)	
Short stature	17 (6.2)	16 (7.0)	1 (2.1)	
Socioeconomic status				0.5
B	58 (21.1)	45 (19.8)	13 (27.1)	
C	114 (41.5)	98 (43.2)	16 (33.3)	
D	54 (19.6)	43 (18.9)	11 (22.9)	
E	49 (17.8)	41 (18.1)	8 (16.7)	
Sleep duration				0.016 *
6 h or less	132 (48.0)	117 (51.5)	15 (31.3)	
7 h	87 (31.6)	64 (28.2)	23 (47.9)	
8 h or more	56 (20.4)	46 (20.3)	10 (20.8)	
Physical activity				0.005 *
High level	54 (19.6)	40 (17.6)	14 (29.2)	
Low level	47 (17.1)	46 (20.3)	1 (2.1)	
Moderate	174 (63.3)	141 (62.1)	33 (68.8)	

Note. BMI = body mass index. Percentages in the “Absence of healthy eating habits” and “Healthy eating habits” columns were calculated within each eating habits group. * *p* < 0.05.

**Table 2:** Unadjusted Analysis of Factors Associated with Healthy Eating Habits.

Characteristic	OR	95% CI	*p*
Sex			
Female	1	Reference	
Male	1.817	0.895–3.762	0.101
School grade			
1st year of secondary school	1	Reference	
2nd year of secondary school	0.346	0.086–1.289	0.122
3rd year of secondary school	0.244	0.049–1.156	0.079
4th year of secondary school	0.133	0.016–1.016	0.057
5th year of secondary school	0.066	0.004–0.862	0.044 *
BMI			
Underweight	1	Reference	
Excess weight	0.484	0.017–13.423	0.626
Normal	0.691	0.025–18.939	0.803
Height-for-age			
Normal	1	Reference	
Short stature	0.59	0.030–3.755	0.636
Socioeconomic status			
B	1	Reference	
C	0.683	0.282–1.680	0.4
D	1.373	0.501–3.780	0.536
E	0.97	0.320–2.851	0.956
Sleep duration			
6 h or less	1	Reference	
7 h	2.652	1.196–6.033	0.018 *
8 h or more	1.115	0.429–2.805	0.818
Physical activity			
High level	1	Reference	
Low level	0.103	0.005–0.630	0.040 *
Moderate	0.883	0.400–2.023	0.763

Note. OR = odds ratio; 95% CI = 95% confidence interval; BMI = body mass index. * *p* < 0.05.

**Table 3:** Multivariable logistic regression model for healthy eating habits among secondary school students.

Variable	B	SE	aOR	95% CI	*p*
Intercept	−1.442	0.577	0.236	0.073–0.707	0.012
Male sex	0.502	0.352	1.653	0.832–3.331	0.154
Grade: 2nd year of secondary school	−0.507	0.548	0.603	0.197–1.731	0.355
Grade: 3rd year of secondary school	−0.44	0.453	0.644	0.264–1.582	0.331
Grade: 4th year of secondary school	−0.65	0.558	0.522	0.167–1.526	0.244
Grade: 5th year of secondary school	−0.895	0.645	0.408	0.103–1.359	0.165
Hours of sleep: 7 h	0.926	0.392	2.525	1.181–5.536	0.018 *
Hours of sleep: 8 h or more	0.182	0.459	1.198	0.474–2.919	0.694
Physical activity: low level	−2.335	1.089	0.097	0.005–0.564	0.032 *
Physical activity: moderate level	−0.178	0.396	0.837	0.390–1.860	0.654

Note. B = beta coefficient; SE = standard error; aOR = adjusted odds ratio; 95% CI = 95% confidence interval. The reference categories were female sex, 1st year of secondary school, sleeping 6 h or less, and high level of physical activity. The adjusted VIF values were sex = 1.050, school grade = 1.015, hours of sleep = 1.028, and physical activity = 1.034, indicating the absence of relevant multicollinearity. * *p* < 0.05.

## Data Availability

The data supporting the findings of this study are not publicly available due to ethical and privacy restrictions, as the study involved minors and includes sensitive sociodemographic, anthropometric, and health-related information. However, anonymized data may be made available by the corresponding author upon reasonable request, subject to approval by the relevant ethics committee and institutional regulations.
